# Protective Effects of Thyme Leaf Extract Against Particulate Matter-Induced Pulmonary Injury in Mice

**DOI:** 10.3390/antiox14111343

**Published:** 2025-11-07

**Authors:** Jae-Kyoung Lee, Khawaja Muhammad Imran Bashir, Hye-Rim Park, Jin-Gwan Kwon, Beom-Rak Choi, Jae-Suk Choi, Sae-Kwang Ku

**Affiliations:** 1 Hongsamdan Co., Ltd., Gongju 32511, Republic of Korea; jklee@hongsamdan.com; 2 Department of Food Regulatory Science, College of Science and Technology, Korea University, Sejong 30019, Republic of Korea; 3 Department of Seafood Science and Technology, The Institute of Marine Industry, Gyeongsang National University, Tongyeong 53064, Republic of Korea; imranbashir@gnu.ac.kr; 4 Nutracore Co., Ltd., Suwon 16514, Republic of Korea; hrpark@nutracore.co.kr (H.-R.P.); jgkwon@nutracore.co.kr (J.-G.K.); brchoi@nutracore.co.kr (B.-R.C.); 5 Department of Anatomy and Histology, College of Korean Medicine, Daegu Haany University, Gyeongsan 38610, Republic of Korea

**Keywords:** antioxidant defense, dexamethasone, expectorant activity, mucus secretion, pulmonary protective effect, respiratory-protective food ingredient, *Thymus vulgaris* L.

## Abstract

Airborne particulate matter (PM), particularly PM_2.5_, contributes to pulmonary injury by inducing oxidative stress and inflammation. Thyme (*Thymus vulgaris* L.) contains bioactive compounds with anti-inflammatory, antioxidant, and expectorant properties. Here, we evaluated the dose-dependent protective effects of thyme extract (TV) against PM_2.5_-induced pulmonary injury in mice, using dexamethasone (DEXA) as a reference anti-inflammatory drug. Subacute pulmonary injury was induced in male Balb/c mice via intranasal administration of PM_2.5_ (1 mg/kg, twice at 48 h intervals). Mice received oral TV (50, 100, or 200 mg/kg) or DEXA (0.75 mg/kg) daily for 10 days. Assessments included lung weight, serum AST/ALT, bronchoalveolar lavage fluid (BALF) leukocyte counts, cytokines (TNF-α, IL-6), chemokines, oxidative stress markers (ROS, lipid peroxidation, antioxidant enzymes), histopathology, and mRNA expression of genes related to inflammation (PI3K/Akt, MAPK, and NF-κB), mucus production (MUC5AC, MUC5B), and apoptosis (Bcl-2, Bax). Exposure to PM_2.5_ caused oxidative stress, pulmonary inflammation, mucus hypersecretion, and histopathological changes. TV treatment dose-dependently reduced leukocyte infiltration, cytokine/chemokine release, ROS generation, and mucus overproduction, while enhancing antioxidant defenses and improving tissue pathology. Effects were comparable but slightly less potent than DEXA. Notably, unlike DEXA, TV reduced mucus hyperplasia and enhanced expectorant activity. No hepatotoxicity was observed. These results indicate that thyme extract could serve as a promising natural candidate for alternative respiratory therapeutics or functional food development.

## 1. Introduction

Air pollution in East Asia, particularly in China, Korea, and Japan, has emerged as a serious public health crisis, largely due to fine dust exposure that threatens both human health and regional climate stability [1,2,3]. Beijing, the capital of China, lies at the heart of this crisis, experiencing large quantities of particulate matter (PM) during spring due to dust storms originating from northwest China, Mongolia, and the Loess Plateau [4]. The situation is further exacerbated by local emissions from industries, vehicular traffic, and coal combustion. Beijing is recognized as one of the most polluted cities globally [5], drawing international attention during the 2008 Olympic Games and prompting the government to implement stricter pollution control measures thereafter [5]. Ground measurements have revealed that the majority of PM has an aerodynamic diameter of 2.5 μm (PM_2.5_), consisting primarily of mineral dust, polycyclic aromatic hydrocarbons (PAHs), and inorganic substances such as sulfates, nitrates, ammonium, and elemental carbon [6]. An excellent overview of PM_2.5_ fluctuations and their environmental consequences in Beijing is provided by Lv et al. [7].

Epidemiological studies have repeatedly shown that PM infiltrates deep in lung tissues, inducing damage and contributing to cardiopulmonary diseases [1,2,3]. Among these, asthma is a chronic inflammatory condition closely linked to air pollutants. According to WHO estimates (2017), approximately 235 million people worldwide are asthmatic, with PM exposure recognized as a key risk factor [8]. Over 70% of inhaled PM deposits below the trachea, with approximately 22% reaching the alveoli [9], thereby elevating oxidative stress in epithelial and lung tissues and inducing inflammation [5,10]. Following exposure, cellular stress markers such as catalase (CAT), superoxide dismutase (SOD), reactive oxygen species (ROS), glutathione peroxidase (GPx), and heme oxygenase-1 (HO-1) are upregulated [11], together with pro-inflammatory cytokines including tumor necrosis factor-α (TNF-α) and interleukin-6 (IL-6) [12]. Schaumann et al. [10] further highlighted the health risks of industrial PM exposure, showing strong associations with childhood asthma and increased lung inflammation.

Among available treatments, dexamethasone (DEXA), a synthetic glucocorticoid with potency 4–5 times higher than prednisolone and 20–30 times greater than hydrocortisone [13], has shown strong anti-inflammatory efficacy in respiratory disorders [14,15] including PM_2.5_-induced pulmonary injury [16]. In earlier studies, water-soluble DEXA administered orally at 0.75 mg/kg was employed as a standard drug for therapeutic evaluation [14,15,16]. In addition, the Balb/c mouse model used in the present study has been extensively validated as an appropriate system for replicating PM_2.5_-induced subacute pulmonary injuries. Earlier studies demonstrated that intranasal instillation of PM_2.5_ reproduces hallmark features of human dust-induced pulmonary disorders, including inflammatory cell infiltration, oxidative stress, airway remodeling, and mucus overproduction [1,2,3]. Thus, this model provides a suitable platform to evaluate preventive and therapeutic interventions against air pollution–induced lung injury.

Given growing environmental and health concerns, there is an urgent need to identify novel preventive and therapeutic strategies against PM-mediated lung injury [2,3]. Medicinal plants offer a promising source of antioxidants and bioactive molecules [17,18], with potential applications in respiratory protection [1,2,3,16]. Thyme (*Thymus vulgaris* L.), a member of the Lamiaceae family native to Southern and Eastern Europe, is one such candidate [19]. Its pharmacological activities, which include antioxidant, anti-inflammatory, antimicrobial, immunomodulatory, and metabolic effects, are largely attributed to its polyphenolic constituents, particularly phenolic acids and flavonoids [20,21,22,23,24,25]. High-performance liquid chromatography (HPLC) analyses of thyme leaf extracts have identified phenolic acids such as rosmarinic acid, methyl rosmarinate, cinnamic acid, caffeic acid, protocatechuic acid, and chlorogenic acid, as well as flavonoids including luteolin, quercetin, apigenin, ferulic acid, zeaxanthin, naringenin, and thymonin [21,26]. Methanolic extracts of thyme leaves have demonstrated antioxidant capacities exceeding those of natural and synthetic antioxidants, including α-tocopherol and BHA [26]. In addition, thyme contains vitamins, particularly C (ascorbic acid), A (retinol), and B6 (pyridoxine, 100 g providing approximately 27% of the daily recommended intake), as well as other minor vitamins such as E, folic acid, and K [27], along with chlorophyll, which may contribute to supportive detoxifying and anticancer effects [20,25].

The present study evaluated the dose-dependent protective effects of thyme extract (TV; 50, 100, and 200 mg/kg) against PM_2.5_-induced pulmonary injury in mice and compared its efficacy with DEXA (0.75 mg/kg). TV was administered orally once daily for 10 days, one hour after each PM_2.5_ intranasal instillation (1 mg/kg at 48 h intervals), to explore its capability as an innovative respiratory protectant and functional food ingredient.

## 2. Materials and Methods

### 2.1. Animal Husbandry

A total of 88 healthy male Balb/cAnNCrlOri (Balb/c) mice (SPF/VAF inbred strain, 6 weeks old at receipt; OrientBio, Seongnam, Republic of Korea) were housed for a 7-day acclimation period before the experiment. All animals used in the study were wild-type with no prior genetic modification or experimental treatment. A total of four mice were placed per polycarbonate cage in a controlled environment (temperature: 20–25 °C; humidity: 30–35%; 12 h light/dark cycle) with ad libitum access to feed (Purinafeed, Seongnam, Republic of Korea) and water. Following acclimatization, the mice were randomly assigned to six groups (*n* = 10 per group; total = 60). Body weights were recorded one day prior to the first PM_2.5_ instillation (intact control: 20.26 ± 0.62 g, range 19.40–21.30 g; PM_2.5_-treated: 20.27 ± 0.78 g, range 18.70–21.80 g). All experimental procedures were performed in accordance with international guidelines for the care and use of laboratory animals and were approved by the Institutional Animal Care and Use Committee of Daegu Haany University, Gyeongsan, Republic of Korea (Approval No. DHU2022-013; approved on 22 February 2022).

Experimental groups were as follows (*n* = 10 per group):

1. Intact control: Distilled water (10 mL/kg, p.o.) + saline (0.1 mL/kg, intranasal)
2. PM_2.5_ control: Distilled water (10 mL/kg, p.o.) + PM_2.5_ (1 mg/kg, intranasal)
3. DEXA: DEXA 0.75 mg/kg (11.40 mg/kg as DEXA-water soluble, p.o.) + PM_2.5_ (1 mg/kg, intranasal)
4. TV_200_: TV 200 mg/kg (p.o.) + PM_2.5_ (1 mg/kg, intranasal)
5. TV_100_: TV 100 mg/kg (p.o.) + PM_2.5_ (1 mg/kg, intranasal)
6. TV_50_: TV 50 mg/kg (p.o.) + PM_2.5_ (1 mg/kg, intranasal)

### 2.2. Induction of Pulmonary Injury by PM_2.5_

Subacute pulmonary injury was induced by intranasal instillation of PM_2.5_ suspensions (NIST 1650b; 10 mg/mL in physiological saline; Sigma-Aldrich, St. Louis, MO, USA). Mice received 0.1 mL/kg (equivalent to 1 mg/kg) twice at 48 h intervals (Day 0 and Day 2), administered 1 h before test article treatment, using micropipettes with yellow tips [1,2,3]. The suspensions were sonicated for 30 min prior to instillation to prevent particle aggregation. Intact control mice received physiological saline (0.1 mL/kg, intranasal) on the same schedule to account for stress associated with intranasal dosing.

### 2.3. Preparations and Administration of Test Articles

The fresh leaf extract of thyme (*Thymus vulgaris* L.; TV extract) was prepared, standardized, and supplied by Plantex (Saint Michel Sur Orge, France) and spray-dried by NUTRACORE (Suwon, Republic of Korea). A detailed extract preparation methodology has been presented in Figure S1. A voucher specimen was deposited in the herbarium of the Medical Research Center for Herbal Convergence on Liver Disease, Daegu Haany University, Gyeongsang, Republic of Korea. In addition, a crude drug reference sample was obtained from the National Institute of Food and Drug Safety Evaluation (NIFDS), Ministry of Food and Drug Safety (MFDS), Republic of Korea. The authenticity of the harvested material was confirmed by comparing its HPLC fingerprint with that of the official reference sample.

The thyme aqueous extract (coded as Batch No. TV-P2118) was a yellowish-brown powder. The extract was prepared in distilled water at concentrations of 20, 10, and 5 mg/mL, which corresponded to doses of 200, 100, and 50 mg/kg body weight for administration. The extract was administered orally by gavage at 10 mL/kg once daily for 10 consecutive days, 1 h after each test article exposure. The highest dose (200 mg/kg) was determined based on preliminary screening results, while the lower doses were established using a twofold serial dilution. Samples were kept at −20 °C until further use.

The dexamethasone-water soluble formulation (Sigma-Aldrich, St. Louis, MO, USA) contained 66 mg/g of dexamethasone. For administration, the white powder was prepared in distilled water at 0.075 mg/mL, equivalent to a dexamethasone dose of 0.75 mg/kg (1.14 mg/mL as DEXA-water soluble). The solution was administered orally at 10 mL/kg once daily for 10 days, 1 h post–PM_2.5_ exposure. The dose level was selected based on previously established anti-inflammatory experimental models [14,15,16]. Dexamethasone solutions were stored at 4 °C until use. For vehicle control groups (intact and PM_2.5_-treated), distilled water was administered in equal volumes to minimize handling-related stress.

### 2.4. Determination of Test Article by HPLC

The quantification of rosmarinic acid present in *Thymus vulgaris* leaf extracts was carried out using an Agilent 1260 Infinity II high-performance liquid chromatography (HPLC) system (Agilent Technologies, Inc., Santa Clara, CA, USA). The instrument was equipped with a UV–Vis detector and a CAPCELL PAK C_18_ UGII column (4.6 mm × 250 mm, 5 μm particle size; CELLACHROM™, Daejeon, Republic of Korea). Both the rosmarinic acid standard and the TV extract were dissolved in methanol and subsequently filtered using a 0.45 μm membrane filter prior to analysis. The chromatographic separation was performed at a column temperature of 30 °C, and rosmarinic acid was monitored at a detection wavelength of 330 nm. The mobile phase consisted of 0.05% trifluoroacetic acid in water (A) and acetonitrile (B). The elution profile was as follows: 0–40 min, 75% A and 25% B (*v*/*v*); 40–50 min: 5% A and 95% B; all gradients were linear. Calibration was performed using rosmarinic acid standard (R4033; Sigma-Aldrich, St. Louis, MO, USA). Each sample was injected at a volume of 10 μL, and the flow rate was maintained at 0.35 mL/min. Quantification was performed by comparing the sample peak areas with those of the standard, and the concentration was determined from the corresponding calibration curve.

### 2.5. Changes in Body Weight

Body weights were measured daily, starting one day before the first PM_2.5_ nasal instillation and oral administration of test articles, and continued for the duration of the experiment using an electronic balance (Precisa Instrument, Dietikon, Switzerland). To reduce individual differences, body weight gain was determined from the day of the first oral administration to 24 h following the 10th administration (Equation (1)).

Body weight gain (g) = Body weight at 24 h after last administration − Body weight at initial PM_2.5_ instillation and test article administration (1)

### 2.6. Serum AST and ALT Assessment

At 24 h following the final administration of test substances, approximately 1 mL of blood was collected from the vena cava under anesthesia using 2–3% isoflurane (Hana Pharm., Hwasung, Republic of Korea) in a gas mixture of 70% N_2_O and 28.5% O_2_, delivered via a rodent inhalation system equipped with a ventilator (Surgivet, Waukesha, WI, USA; Harvard Apparatus, Cambridge, UK). Blood samples were centrifuged at 12,500 rpm for 10 min at 4 °C in clot-activated serum tubes (Gyrozen, Daejeon, Republic of Korea), and serum was stored at −150 °C (Sanyo, Tokyo, Japan) until analysis. Levels of aspartate transaminase (AST) and alanine transaminase (ALT) were measured in IU/L using an automated analyzer (Dri-Chem NX500i, Fuji Medical Systems, Tokyo, Japan).

### 2.7. Lung Weight Measurement

Lungs were excised under inhalation anesthesia 24 h after the last treatment and weighed on an electronic balance (Precisa Instrument, Dietikon, Switzerland). Absolute wet lung weights (g) were recorded, and relative lung weights (% of body weight) were calculated using Equation (2) to account for inter-individual differences:

Relative lung weight (%) = (Absolute lung wet weight/Body weight at sacrifice) × 100(2)

### 2.8. Lung Collection and Gross Examination

Following lung excision, the left secondary bronchus and right lower secondary bronchus were ligated using 3-0 nylon (AILEE, Busan, Republic of Korea). Lobar allocation was as follows: right upper and middle lobes for bronchoalveolar lavage fluid (BALF) collection; right lower lobes for assays of matrix metalloproteinases (MMPs), substance P, acetylcholine (ACh), ROS, lipid peroxidation, antioxidant enzymes, and cytokines; and left lobes for gross morphological evaluation, histopathology, and real-time RT-PCR. Gross morphology was photographed with a digital camera (FinePix S700; Fujifilm, Tokyo, Japan), and the proportion of congested areas (%) was quantified from images using an automated image analyzer (iSolution FL v9.1; IMT i-Solution, Burnaby, BC, Canada).

### 2.9. BALF Collection and Cytology

After ligation, 1 mL of physiological saline was instilled via a 20 G tracheal cannula and aspirated; this process was repeated twice per animal. BALF samples were pooled, as previously described [14,28] with minor modifications. Total cell counts were determined using an automated cell counter (Countess C10281; Invitrogen, Carlsbad, CA, USA) with trypan blue exclusion. Leukocyte differentials (lymphocytes, neutrophils, eosinophils, monocytes) were obtained using a hematology analyzer (Cell-DYN 3700; Abbott Laboratories, Abbott Park, IL, USA).

### 2.10. Lung Homogenate Preparation

Right lower lung lobes were homogenized in normal saline using a bead beater (Taco™Pre; GeneResearch Biotechnology, Taichung, Taiwan) and an ultrasonic disruptor (Madell Technology, Ontario, CA, USA). Homogenates were stored at −150 °C until further analyses.

### 2.11. Quantification of Cytokines, MMP, Substance P and ACh

Lung homogenates were centrifuged at 12,500 rpm for 30 min at 4 °C (Gyrozen, Daejeon, Republic of Korea). Supernatants were analyzed for TNF-α, IL-6, chemokines (CXCL-1, CXCL-2), MMP-9, MMP-12, substance P, and ACh using ELISA kits (MyBioSource, San Diego, CA, USA), following manufacturer instructions. Absorbance was read at 450 nm using a microplate reader (Sunrise, Tecan, Männedorf, Switzerland).

### 2.12. Lipid Peroxidation Assay

Lung homogenates were centrifuged at 12,000× *g* for 15 min at 4 °C as described by Kavutcu et al. [29]. Lipid peroxidation was determined by measuring malondialdehyde (MDA) levels using the thiobarbituric acid assay, with absorbance at 525 nm (OPTIZEN POP, Mecasys, Daejeon, Republic of Korea), and expressed as nM MDA per mg protein [30]. Protein concentrations were determined by the Lowry method [31] using BSA as a standard.

### 2.13. ROS Measurement

ROS levels in lung homogenates were quantified using the DCFDA probe (Abcam, Cambridge, MN, USA). Fluorescence intensity was measured at 490/520 nm (Versa-Max™; Molecular Devices, Sunnyvale, CA, USA) and normalized to protein content, expressed as RFU/μg protein [32].

### 2.14. Assessment of Antioxidant Enzymes

Lung homogenates were mixed with 25% trichloroacetic acid (Merck, West Point, CA, USA) and centrifuged at 4200 rpm for 40 min at 4 °C. Glutathione (GSH) content was measured at 412 nm using 2-nitrobenzoic acid [33]. CAT activity was measured at 240 nm via H_2_O_2_ decomposition [34], and SOD activity was assessed by inhibition of nitroblue tetrazolium reduction [35], read at 560 nm. Enzyme activities were expressed as U/mg protein. All reagents were obtained from Sigma-Aldrich (St. Louis, MO, USA).

### 2.15. Real-Time RT-PCR

The mRNA expression of mucins (MUC5AC, MUC5B), nuclear factor kappa-light-chain-enhancer of activated B cells (NF-κB), p38 mitogen-activated protein kinase (p38 MAPK), phosphatase and tensin homolog (PTEN), phosphoinositide 3-kinase (PI3K), protein kinase B (Akt), B-cell leukemia/lymphoma 2 (Bcl-2), and BCL2 associated x (Bax) gene was measured using real-time RT-PCR [36,37]. RNA was extracted using Trizol (Invitrogen, Carlsbad, CA, USA), treated with DNase I (Thermo Fisher Scientific, Rockford, IL, USA), and reverse transcribed with a High-Capacity cDNA Kit (Thermo Fisher Scientific). PCR amplification was performed on an ABI StepOnePlus system (50 cycles: 95 °C for 1 min; 95 °C for 15 s; 55–65 °C for 20 s; 72 °C for 30 s). β-actin was used as a reference gene, and relative expression was calculated using the 2^−ΔΔCq^ method [38]. Primer sequences are listed in Table S1.

### 2.16. Histopathology

Left lung lobes were fixed in 10% neutral-buffered formalin for 24 h, embedded in paraffin, and sectioned at 3–4 μm. Sections were stained with hematoxylin and eosin (H&E) for general morphology and periodic acid–Schiff (PAS) for mucus-producing cells [14,39,40]. Slides were examined under light microscopy (Eclipse 80i; Nikon, Tokyo, Japan) with imaging via ProgRes™ C5 camera (Jenoptik, Jena, Germany) and analyzed using iSolution FL software (v9.1). Parameters measured included alveolar surface area, septal thickness, inflammatory cell density, PAS-positive goblet cell counts, and the percentage of PAS-positive cell areas. At least 10 fields per group were evaluated in a blinded manner.

### 2.17. Statistical Analysis

Data are presented as mean ± SD (*n* = 10). Levene’s test was used to assess homogeneity of variance. Datasets with equal variance were analyzed by one-way ANOVA followed by Tukey’s HSD test, while datasets with unequal variance were analyzed using Dunnett’s T3 test [41,42]. Statistical significance was set at *p* < 0.05. Analyses were conducted using SPSS v18.0 (SPSS Inc., Chicago, IL, USA). Percentage changes relative to vehicle control or PM_2.5_ control were calculated using Equations (3) and (4) [14,28,40]:

% change vs. vehicle control = [(PM_2.5_ control − vehicle control)/vehicle control] × 100 (3)

% change vs. PM_2.5_ control = [(Treatment − PM_2.5_ control)/PM_2.5_ control] × 100 (4)

## 3. Results

### 3.1. Rosmarinic Acid Content in TV Extract

**Figure 1:**
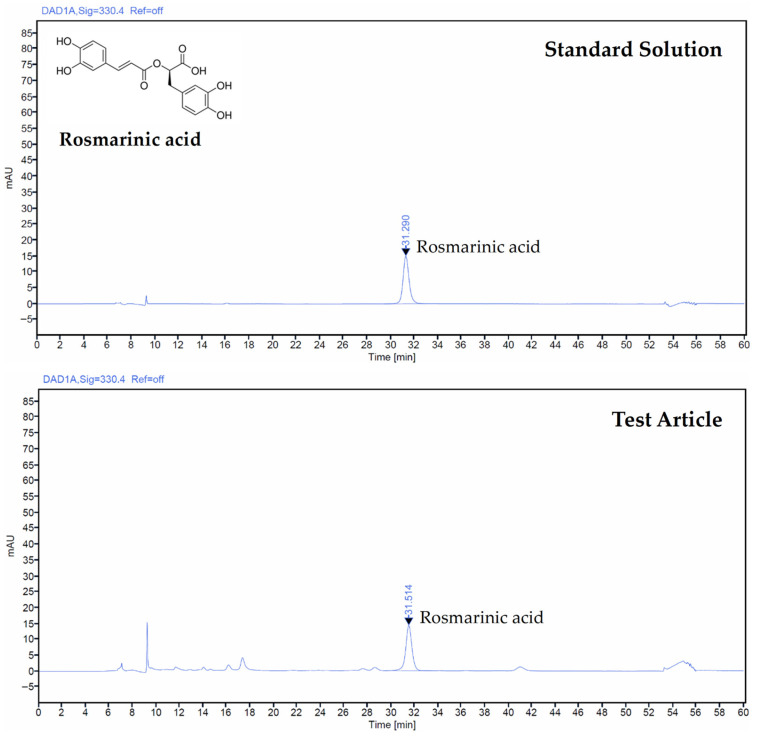
HPLC chromatograms of rosmarinic acid in the standard solution and the *Thymus vulgaris* L. leaf extract (TV extract).

HPLC analysis determined that the concentration of rosmarinic acid in the TV extract was 7.2 mg/g. Quantification was performed by comparing the relative peak area of the extract to that of the standard solution, as shown in Figure 1.

### 3.2. Body Weight Changes

**Figure 2:**
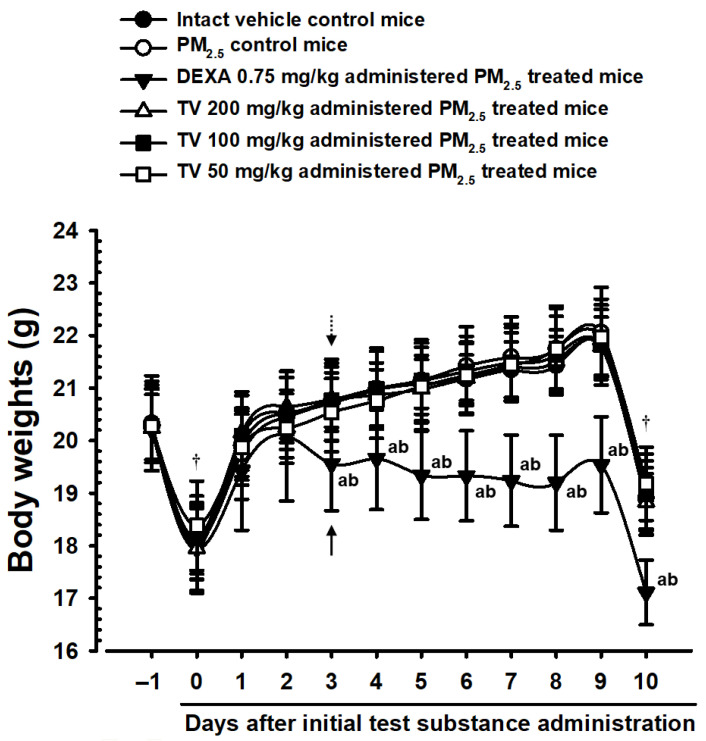
Body weight trends in intact or PM_2.5_-treated pulmonary-injured mice. Values represent means ± SD for 10 mice per group. PM_2.5_ refers to Diesel Particulate Matter NIST 1650b; DEXA indicates dexamethasone treatment; TV refers to thyme (*Thymus vulgaris* L.) leaf extract; THSD denotes Tukey’s Honest Significant Difference test. Body weights were monitored from one day prior to the first administration (Day-1) until 24 h after the 10th administration (Day-10). All mice were fasted overnight before the initial administration and at sacrifice. Arrows in the figure indicate significant decreases in body weight following DEXA administration (0.75 mg/kg) relative to intact vehicle control (†), and dot arrows indicate reductions compared with PM_2.5_ control mice. Significant differences are indicated as follows: ^a^ *p* < 0.01 vs. intact vehicle control (THSD test); ^b^ *p* < 0.01 vs. PM_2.5_ control (THSD test).

Body weights or gains did not show a significant change in PM_2.5_-treated mice compared with intact vehicle controls over the experimental period of 10 days, except for DEXA 0.75 mg/kg-treated mice, which showed significant decreases in body weight (*p* < 0.01) from day 3 and reduced weight gains throughout the experimental period (Table S2 and Figure 2). TV treatment at 200, 100, and 50 mg/kg did not significantly alter body weights or gains compared with PM_2.5_ controls. Body weight gains in PM_2.5_ controls changed by 1.23% relative to intact controls, whereas changes in DEXA and TV-treated groups were −202.44%, 7.32%, 1.22%, and −2.44%, respectively.

### 3.3. Gross Lung Inspections and Weights

**Figure 3:**
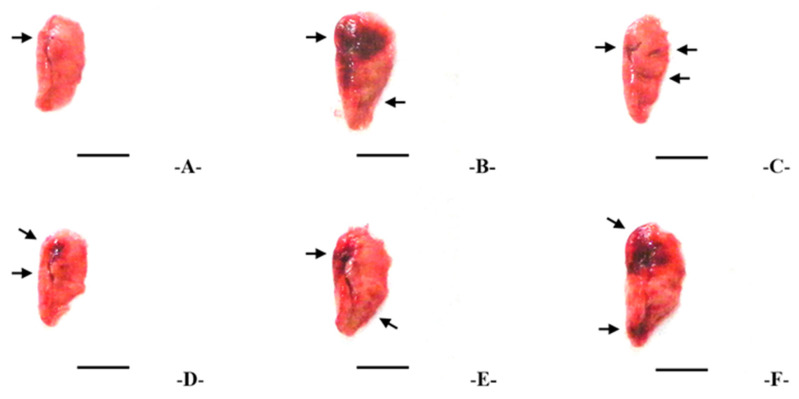
Representative gross lung images showing congestion in left lung lobes of each treatment group. Treatments are designated as follows: (**A**)—intact vehicle control (distilled water orally, saline intranasal), (**B**)—PM_2.5_ control (distilled water orally, PM_2.5_ intranasal), (**C**)—DEXA (0.75 mg/kg orally, PM_2.5_ intranasal), (**D**)—TV_200_ (200 mg/kg TV orally, PM_2.5_ intranasal), (**E**)—TV_100_ (100 mg/kg TV orally, PM_2.5_ intranasal), (**F**)—TV_50_ (50 mg/kg TV orally, PM_2.5_ intranasal). PM_2.5_ refers to Diesel Particulate Matter NIST 1650b; DEXA indicates dexamethasone treatment; TV refers to thyme (*Thymus vulgaris* L.) leaf extract; Congested regions are highlighted with arrows. Scale bars = 6.00 mm.

PM_2.5_ exposure induced notable lung focal congestion and enlargement, with significant increases (*p* < 0.01) in absolute, and relative lung weights, and gross congestional area compared with intact controls (Table S3 and Figure 3). Oral TV treatment dose-dependently reduced these parameters, with inhibitory effects slightly lower than DEXA 0.75 mg/kg. Gross congestional areas in PM_2.5_ controls increased by 3770.21%, while DEXA, TV 200, 100, and 50 mg/kg treatments reduced this by −87.72%, −75.60%, −62.46%, and −40.09%, respectively. Similar trends were observed for absolute and relative lung weights.

### 3.4. BALF Cytology

PM_2.5_ exposure caused marked pulmonary inflammation, with notable increase (*p* < 0.01) in BALF total cells, leukocytes, eosinophils, neutrophils, lymphocytes, and monocytes compared with intact controls (Table 1). Total cell numbers increased by 715.63%, total leukocytes by 777.05%, lymphocytes by 908.82%, neutrophils by 750.43%, eosinophils by 13,354.55%, and monocytes by 404.62%. Oral supply of TV (200—50 mg/kg) significantly and dose-dependently inhibited these elevations, demonstrating strong anti-inflammatory activity, although slightly lower than DEXA 0.75 mg/kg. This indicates that TV effectively mitigates PM_2.5_-induced immune cell recruitment and pulmonary inflammatory responses.

### 3.5. Changes in AST and ALT Levels

**Figure 4:**
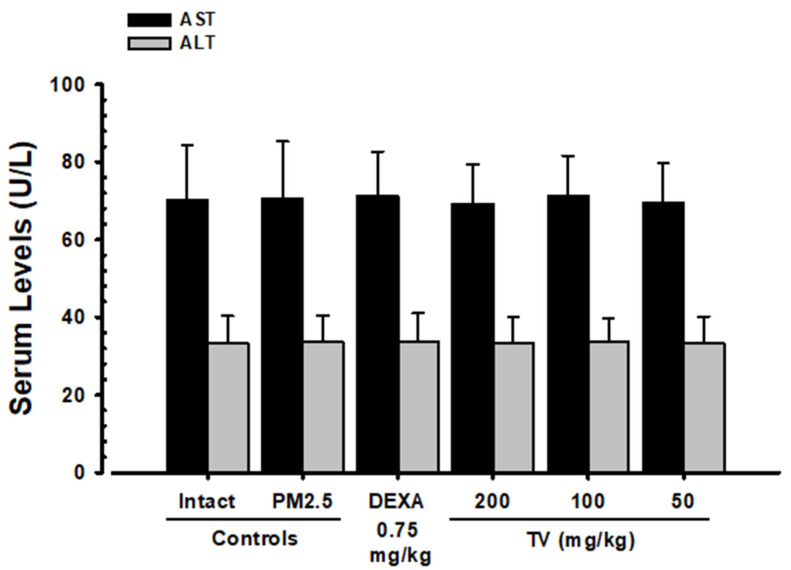
Serum AST and ALT levels in intact and PM_2.5_-exposed mice. Values represent means ± SD for 10 mice per group. PM_2.5_ refers to Diesel Particulate Matter NIST 1650b; DEXA indicates dexamethasone treatment; TV refers to thyme (*Thymus vulgaris* L.) leaf extract; AST refers to aspartate aminotransferase; ALT indicates alanine aminotransferase.

No markable differences in serum AST and ALT amounts were observed in PM_2.5_-exposed mice or any treatment group, suggesting that neither PM_2.5_ exposure nor TV administration caused hepatotoxicity under the study conditions (Figure 4). AST changes were minimal (0.57% in PM_2.5_ control vs. intact control), and ALT changes were similarly negligible (0.60%), confirming systemic safety of TV treatments.

### 3.6. Lung Cytokine Levels

PM_2.5_ exposure significantly elevated pro-inflammatory cytokines: IL-6 (1431.86%), TNF-α (683.29%), CXCL1 (1003.30%), and CXCL2 (1058.36%) compared with intact controls (Table 2). TV treatment dose-dependently reduced these elevations, indicating suppression of local pulmonary inflammation. For instance, TNF-α levels decreased by 62.32%, 53.44%, and 40.83% in TV 200, 100, and 50 mg/kg groups, respectively, while DEXA achieved a 71.43% reduction. These data highlight TV’s potential to modulate cytokine-mediated inflammatory signaling pathways in PM_2.5_-induced lung injury.

### 3.7. MMP-9 and MMP-12 Contents

**Figure 5:**
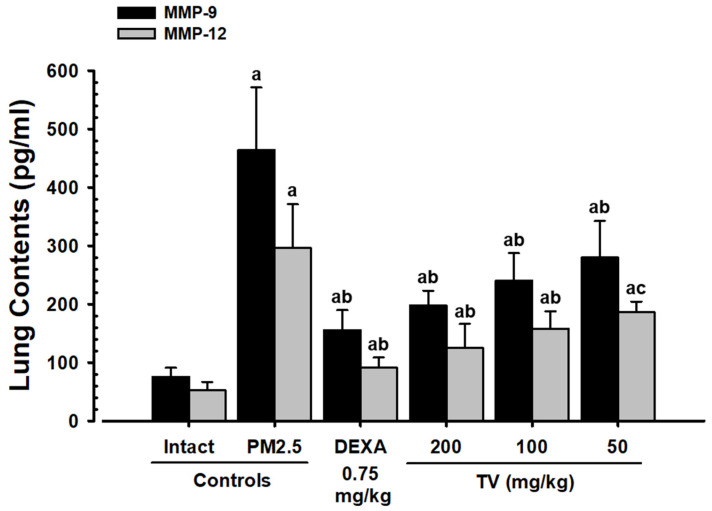
Lung MMP content in intact or PM_2.5_-treated mice. Values represent means ± SD for 10 mice per group. PM_2.5_ refers to Diesel Particulate Matter NIST 1650b; DEXA indicates dexamethasone treatment; TV refers to thyme (*Thymus vulgaris* L.) leaf extract; MMP indicates matrix metalloproteinase; DT3 refers to Dunnett’s T3 test. Statistical significance is indicated as follows: ^a^ *p* < 0.01 vs. intact vehicle; ^b^ *p* < 0.01, ^c^ *p* < 0.05 vs. PM_2.5_ control (DT3 test).

Noticeable elevations in PM_2.5_-induced lung MMP-9 (511.48%) and MMP-12 (467.50%) were observed, reflecting extracellular matrix remodeling and potential tissue damage (Figure 5). TV treatment dose-dependently reduced MMP-9 by 57.32%, 48.19%, and 39.66%, and MMP-12 by 57.65%, 46.61%, and 37.16% at 200, 100, and 50 mg/kg, respectively. These findings suggest that TV may protect against PM_2.5_-induced lung tissue remodeling.

### 3.8. Lung Substance P and ACh Content

**Figure 6:**
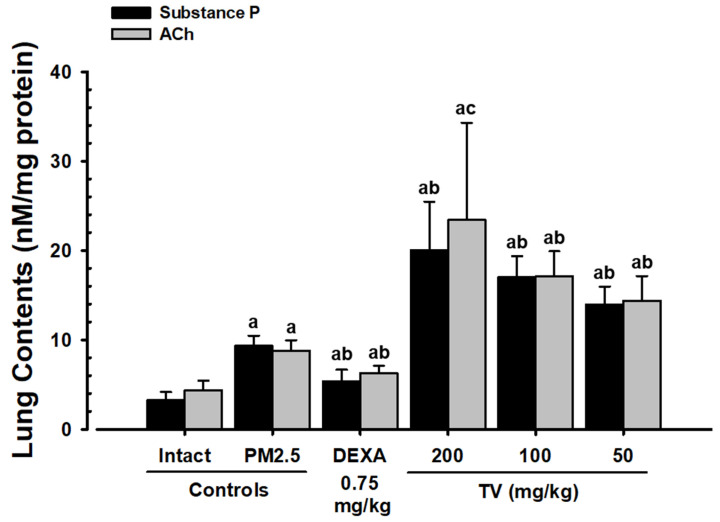
Substance P and ACh content in intact or PM_2.5_-treated mice. Values represent means ± SD for 10 mice per group. PM_2.5_ refers to Diesel Particulate Matter NIST 1650b; DEXA indicates dexamethasone treatment; TV refers to thyme (*Thymus vulgaris* L.) leaf extract; Ach indicates acetylcholine; DT3 refers to Dunnett’s T3 test. Statistical significance is indicated as follows: ^a^ *p* < 0.01 vs. intact vehicle; ^b^ *p* < 0.01, ^c^ *p* < 0.05 vs. PM_2.5_ control (DT3 test).

PM_2.5_ exposure markedly increased lung substance P (187.12%) and ACh (101.37%), contributing to airway hyperreactivity and mucus secretion (Figure 6). DEXA significantly reduced these levels (−42.16% and −28.50%, respectively). Interestingly, TV treatment dose-dependently increased substance P (49.65–115.24%) and ACh (63.55–166.24%), suggesting a modulatory effect on pulmonary neuroimmune signaling that may support mucus clearance and bronchodilation.

### 3.9. Lung Lipid Peroxidation and Antioxidant Defense

PM_2.5_ caused pronounced oxidative stress, reflected by elevated MDA (287.91%) and ROS (441.81%) levels, along with depletion of antioxidant defenses (GSH −85.02%, SOD −79.77%, CAT −85.31%). TV treatment dose-dependently suppressed ROS and MDA levels while restoring GSH content and SOD/CAT activities, highlighting its potent antioxidant activity (Table 3). The effects were slightly lower than DEXA but clearly protective against PM_2.5_-induced oxidative lung injury.

### 3.10. Mucus Production Genes

Significant upregulation of MUC5AC (396.59%) and MUC5B (183.86%) mRNA was observed in PM_2.5_ controls, consistent with hypersecretory airway responses (Table 4). TV treatment dose-dependently suppressed these expressions, suggesting a regulatory effect on mucus production and potential improvement of airway clearance. DEXA exhibited slightly stronger inhibitory effects but did not enhance expectoration-related features, unlike TV.

### 3.11. Oxidative Stress- and Inflammation-Related Genes

PM_2.5_ exposure significantly increased NF-κB (763.66%), p38 MAPK (625.95%), PI3K (534.23%), and Akt (408.69%) mRNA expressions while decreasing PTEN (−72.60%), reflecting heightened oxidative stress and inflammatory signaling (Table 4). TV treatment dose-dependently reversed these changes, indicating suppression of pro-inflammatory pathways and partial restoration of protective PTEN signaling. These results suggest TV modulates critical molecular pathways underlying PM_2.5_-induced pulmonary injury.

### 3.12. Apoptosis-Related Genes

PM_2.5_ induced a pro-apoptotic shift with decreased Bcl-2 (−62.19%) and increased Bax (590.04%) mRNA expression (Table 4). TV treatment dose-dependently normalized these expressions, demonstrating anti-apoptotic effects and potential protection of alveolar epithelial integrity.

### 3.13. Lung Histopathology

**Figure 7:**
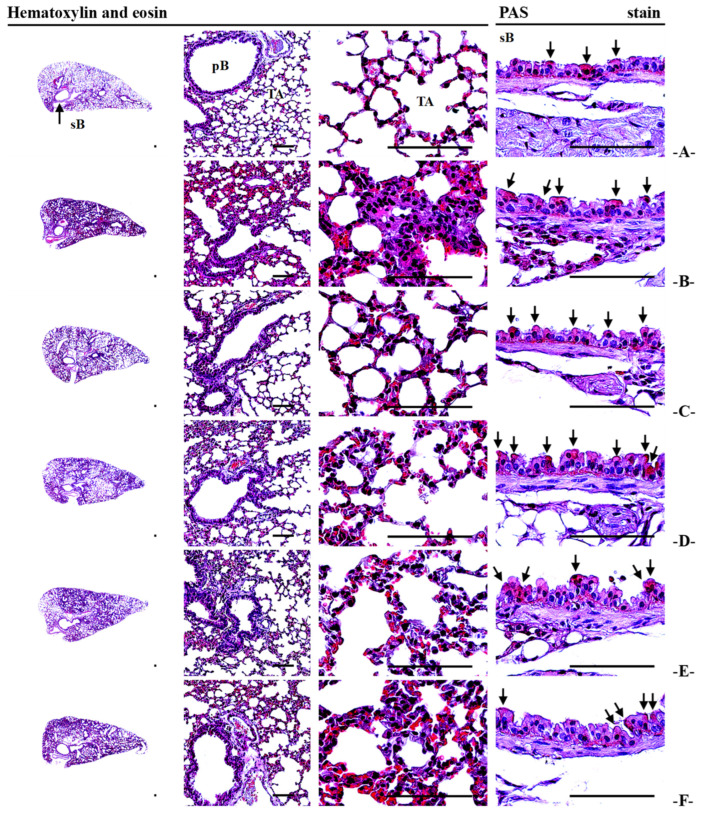
Representative histopathological profiles of left lung lobes in intact and PM_2.5_-treated mice. Treatments are designated as follows: (**A**)—intact vehicle control (distilled water orally, saline intranasal), (**B**)—PM_2.5_ control (distilled water orally, PM_2.5_ intranasal), (**C**)—DEXA (0.75 mg/kg orally, PM_2.5_ intranasal), (**D**)—TV_200_ (200 mg/kg TV orally, PM_2.5_ intranasal), (**E**)—TV_100_ (100 mg/kg TV orally, PM_2.5_ intranasal), (**F**)—TV_50_ (50 mg/kg TV orally, PM_2.5_ intranasal). PM_2.5_ refers to Diesel Particulate Matter NIST 1650b; DEXA indicates dexamethasone treatment; TV refers to thyme (*Thymus vulgaris* L.) leaf extract; ASA stands for alveolar surface area; PAS indicates periodic acid Schiff; sB denotes secondary bronchus; pB refers to primary bronchiole; TA stands for terminal respiratory bronchiole-alveoli. PAS+ mucus-producing cells are highlighted with arrows. Scale bars = 200 µm.

Histopathological analysis confirmed PM_2.5_-induced lung injury, including alveolar septal thickening, inflammatory cell infiltration, and hyperplasia of PAS+ mucus-producing cells, resulting in decreased alveolar surface area (−53.39%). TV treatment dose-dependently reduced septal thickening and inflammatory infiltration, partially restored ASA, and increased PAS+ cell numbers, their occupied percentages, and secondary bronchus mucosa thickness, suggesting both protective and expectorant effects (Figure 7; Table 5). DEXA mainly reduced inflammation but did not affect mucus hyperplasia, highlighting the complementary benefit of TV in maintaining airway clearance.

## 4. Discussion

Particulate matter has emerged as a major environmental health issue due to its profound effects on human respiratory and systemic health. Among its fractions, fine particles < 2.5 μm in diameter (PM_2.5_) are particularly dangerous, as they can penetrate deep into the lung, accumulate in the alveoli, and evade mucociliary clearance [43,44,45,46]. The harmful effects of PM_2.5_ are further amplified by adsorbed toxic substances such as organic compounds, heavy metals, and sulfates, which enhance oxidative stress and inflammatory signaling in pulmonary tissues [47,48]. Epidemiological studies have linked chronic PM_2.5_ exposure to a spectrum of health-related problems, including cardiovascular disorders, lung cancer, chronic obstructive pulmonary disease (COPD), and asthma, highlighting the critical necessity for both preventive and treatment strategies [49,50].

In this study, repeated intranasal instillation of PM_2.5_ in Balb/c mice induced classic features of pulmonary injury, including lung congestion, increased lung weight, and leukocyte infiltration in BALF. In parallel, there was upregulation of chemokines (CXCL1, CXCL2) and inflammatory cytokines (IL-6, TNF-α), as well as increased expression of matrix metalloproteinases (MMP-9, MMP-12), which contribute to extracellular matrix degradation and airway remodeling [51,52]. PM_2.5_ also triggered oxidative stress, evidenced by elevated ROS and lipid peroxidation, alongside depletion of endogenous antioxidant systems such as CAT, GSH, and SOD. These results support previous studies indicating that oxidative stress plays a key role in mediating PM_2.5_-induced lung toxicity [53,54,55].

Treatment with *T. vulgaris* (TV) extract demonstrated robust protective effects across multiple pathological dimensions. TV restored antioxidant defenses by replenishing GSH, SOD, and CAT levels while reducing ROS and MDA accumulation, highlighting its potent free-radical scavenging activity. This effect is likely attributable to bioactive constituents such as rosmarinic acid, which has well-documented antioxidant properties [56,57,58]. In parallel, TV suppressed key inflammatory signaling pathways, including p38 MAPK, PI3K/Akt, and NF-κB, while restoring PTEN expression, thereby reducing the transcription of chemokines and pro-inflammatory genes. Consequently, TV significantly decreased BALF leukocyte infiltration and systemic pulmonary inflammation, aligning with earlier studies on the anti-inflammatory activity of thyme and its bioactive components [19,21,22,23,59,60,61].

PM_2.5_ exposure also induced apoptotic imbalance, with decreased Bcl-2 and increased Bax expression, contributing to epithelial cell death and alveolar structural damage. TV treatment effectively restored this apoptotic balance, enhancing Bcl-2 and reducing Bax expression, thus preserving alveolar integrity. Histopathological evaluation further confirmed that TV reduced alveolar septal thickening, inflammatory infiltration, and mucus cell hyperplasia, demonstrating comprehensive tissue-level protection.

A particularly notable finding was the mucoregulatory effect of TV. PM_2.5_ exposure enhanced expression of mucin-related genes (MUC5AC, MUC5B), as well as secretagogue substances P and ACh, promoting mucus hypersecretion and impaired airway clearance. TV extract dose-dependently suppressed these changes, reducing alveolar septal thickness and mucin-related gene expressions while facilitating airway clearance—increasing P and ACh contents and PAS+ cell proliferation and hypertrophy. This dual action—limiting excessive mucus while enhancing expectoration—is clinically significant for respiratory diseases such as COPD and asthma, where impaired mucociliary clearance exacerbates pathology [62]. However, future studies should include physiological measurements of substance P and ACh to better elucidate the relationship between the effects of the TV extract and the modulation of bronchiolar hyperresponsiveness, with mucin distributions throughout airways.

Comparison with DEXA (0.75 mg/kg) revealed important therapeutic distinctions. While DEXA strongly suppressed inflammation, it did not fully mitigate mucus cell hyperplasia or bronchial thickening, and systemic adverse effects, including reductions in body weight, were noted. In contrast, TV exhibited broader pulmonary protection by combining anti-inflammatory, antioxidant, anti-apoptotic, and mucoregulatory effects without adverse systemic outcomes, highlighting its potential as a safer, multi-target therapeutic strategy. Collectively, these findings suggest that TV confers comprehensive protection against PM_2.5_-induced pulmonary injury. By simultaneously targeting oxidative stress, inflammation, apoptosis, and mucus hypersecretion, TV provides a broader protective profile than conventional anti-inflammatory drugs, supporting its prospective role as a natural therapeutic or functional food candidate for protecting respiratory function.

Nevertheless, several limitations should be acknowledged. This study employed a subacute 10-day PM_2.5_ exposure model, providing valuable insights into short-term pulmonary responses, but it may not fully capture the complexity of chronic human exposure. While the current findings suggest a regulatory effect of TV extract on airway mediators such as substance P and ACh, complementary physiological assessments of bronchiolar responsiveness would further strengthen the mechanistic understanding of these effects. Phytochemical characterization in this study focused on rosmarinic acid as a representative marker; however, identification and quantification of other polyphenolic constituents present in TV extract warrant further investigation. A more comprehensive phytochemical analysis using advanced techniques such as UHPLC-ESI-MS or HPLC-PDA would enable a clearer delineation of the bioactive components responsible for the observed effects. Future studies could also explore chronic exposure models, include pharmacokinetic evaluations of individual constituents, and perform comparative analyses with standard therapeutic agents such as mucolytics and bronchodilators to better define the clinical relevance and translational potential of *T. vulgaris* leaf extract.

## 5. Conclusions

The present study demonstrated that oral administration of standardized *T. vulgaris* extract (TV extract at 200—50 mg/kg) provided dose-dependent protective effects under conditions of PM_2.5_-induced subacute pulmonary damage in Balb/c mice. TV significantly reduced oxidative stress, inflammatory cytokines, MMP activity, and apoptosis, while restoring antioxidant defenses and normalizing PI3K/Akt and p38 MAPK signaling. Unlike dexamethasone (DEXA, 0.75 mg/kg), which exhibited stronger anti-inflammatory effects, TV additionally exerted unique mucolytic–expectorant activities by increasing substance P and ACh levels, reducing MUC5AC and MUC5B expression, and alleviating PAS+ cell hyperplasia and mucosal thickening, thereby promoting mucus clearance. Histopathology and gross examinations confirmed that TV alleviated PM_2.5_-induced lung congestion, cellular infiltration, and septal thickening, while serum AST and ALT analysis indicated no hepatotoxicity. Collectively, these findings suggest that although TV was slightly less potent than DEXA in suppressing inflammation, its combined antioxidant, anti-inflammatory, anti-apoptotic, and mucoregulatory actions, together with a favorable safety profile, highlight its efficacy as a natural respiratory-protective agent and functional food ingredient against PM_2.5_-induced pulmonary injury.

## Figures and Tables

**Table 1:** Cytological analysis of BALF in intact or PM_2.5_-treated pulmonary-injured mice.

Groups	Total Cells	Total Leukocytes	Differential Counts			
			Lymphocytes	Neutrophils	Eosinophils	Monocytes
Controls						
Intact vehicle	9.60 ± 2.95	6.10 ± 1.73	3.40 ± 1.17	1.15 ± 0.34	0.01 ± 0.01	1.30 ± 0.87
PM_2.5_	78.30 ± 12.36 ^c^	53.50 ± 13.18 ^c^	34.30 ± 10.08 ^c^	9.78 ± 2.82 ^c^	1.48 ± 0.23 ^c^	6.56 ± 1.43 ^a^
Reference						
DEXA	18.60 ± 3.72 ^cd^	11.60 ± 2.41 ^cd^	7.30 ± 2.50 ^cd^	2.13 ± 0.40 ^cd^	0.04 ± 0.03 ^d^	1.95 ± 0.68 ^b^
Test article–TV						
200 mg/kg	33.80 ± 10.49 ^cd^	19.50 ± 4.86 ^cd^	12.70 ± 3.20 ^cd^	3.79 ± 1.11 ^cd^	0.17 ± 0.08 ^cd^	2.65 ± 0.76 ^b^
100 mg/kg	44.30 ± 6.70 ^cd^	24.40 ± 3.84 ^cd^	15.20 ± 2.94 ^cd^	4.44 ± 0.80 ^cd^	0.67 ± 0.27 ^cd^	3.54 ± 1.09 ^ab^
50 mg/kg	54.10 ± 7.55 ^cd^	29.50 ± 6.45 ^cd^	18.80 ± 4.98 ^ce^	5.38 ± 1.33 ^cd^	0.85 ± 0.22 ^cd^	4.33 ± 1.19 ^ab^

Values represent means ± SD for 10 mice per group. Unit: ×10^4^ cells/mL; PM_2.5_ refers to Diesel Particulate Matter NIST 1650b; DEXA indicates dexamethasone treatment; TV refers to thyme (*Thymus vulgaris* L.) leaf extract; BALF: Bronchoalveolar lavage fluid; THSD denotes Tukey’s Honest Significant Difference test; DT3 indicates Dunnett’s T3 test. Statistical significance is indicated as follows: ^a,c^ *p* < 0.01 vs. intact vehicle control; ^b,d,e^ *p* < 0.01 or *p* < 0.05 vs. PM_2.5_ control, depending on THSD or Dunnett’s T3 tests.

**Table 2:** Lung cytokine content in intact or PM_2.5_-treated mice.

Groups	Lung Contents (pg/mL)			
	TNF-α	IL-6	CXCL1	CXCL2
Controls				
Intact vehicle	29.21 ± 8.72	27.88 ± 11.74	30.37 ± 10.35	16.88 ± 4.30
PM_2.5_	228.82 ± 51.32 ^a^	427.08 ± 54.81 ^a^	340.54 ± 85.86 ^a^	195.53 ± 26.07 ^a^
Reference				
DEXA	65.37 ± 16.34 ^ab^	75.42 ± 13.54 ^ab^	113.70 ± 29.25 ^ab^	61.49 ± 20.30 ^ab^
Test article—TV				
200 mg/kg	86.22 ± 17.23 ^ab^	130.08 ± 45.34 ^ab^	156.66 ± 28.26 ^ab^	89.69 ± 14.28 ^ab^
100 mg/kg	106.55 ± 19.58 ^ab^	197.58 ± 57.64 ^ab^	187.38 ± 29.10 ^ab^	109.88 ± 18.15 ^ab^
50 mg/kg	135.41 ± 18.90 ^ab^	262.57 ± 52.84 ^ab^	210.09 ± 35.64 ^ac^	124.70 ± 23.26 ^ab^

Values represent means ± SD for 10 mice per group. PM_2.5_ refers to Diesel Particulate Matter NIST 1650b; DEXA indicates dexamethasone treatment; TV refers to thyme (*Thymus vulgaris* L.) leaf extract; TNF refers to tumor necrosis factor; IL indicates interleukin; CXCL refers to the chemokine (C-X-C motif) ligand; THSD denotes Tukey’s Honest Significant Difference test; DT3 indicates Dunnett’s T3 test. Statistical significance is indicated as follows: ^a^ *p* < 0.01 vs. intact vehicle; ^b^ *p* < 0.01, ^c^ *p* < 0.05 vs. PM_2.5_ control.

**Table 3:** Lung MDA, GSH content, and CAT/SOD activities in intact or PM_2.5_-treated mice.

Groups	Lung Contents (nM/mg Protein)			Lung Enzyme Activity (U/mg Protein)	
	MDA	ROS	GSH	SOD	CAT
Controls					
Intact vehicle	3.56 ± 1.34	23.31 ± 10.58	41.96 ± 12.99	322.80 ± 47.80	70.10 ± 13.24
PM_2.5_	19.31 ± 2.80 ^a^	90.43 ± 10.05 ^a^	6.29 ± 1.93 ^d^	65.30 ± 19.27 ^d^	10.30 ± 2.11 ^d^
Reference					
DEXA	6.75 ± 1.70 ^bc^	41.35 ± 10.05 ^ac^	19.17 ± 3.97 ^de^	188.60 ± 41.95 ^de^	38.20 ± 12.77 ^de^
Test article—TV					
200 mg/kg	8.92 ± 2.44 ^ac^	48.72 ± 11.29 ^ac^	15.95 ± 2.47 ^de^	166.10 ± 27.13 ^de^	30.80 ± 10.78 ^de^
100 mg/kg	10.70 ± 2.18 ^ac^	59.25 ± 10.12 ^ac^	13.95 ± 2.21 ^de^	145.70 ± 29.81 ^de^	23.20 ± 7.71 ^de^
50 mg/kg	12.36 ± 1.72 ^ac^	64.21 ± 12.31 ^ac^	12.47 ± 2.87 ^de^	120.00 ± 14.91 ^de^	18.90 ± 5.26 ^de^

Values represent means ± SD for 10 mice per group. PM_2.5_ refers to Diesel Particulate Matter NIST 1650b; DEXA indicates dexamethasone treatment; TV refers to thyme (*Thymus vulgaris* L.) leaf extract; MDA refers to malondialdehyde; ROS indicates reactive oxygen species; GSH means glutathione; CAT refers to catalase; SOD stands for superoxide dismutase; THSD denotes Tukey’s Honest Significant Difference test; DT3 indicates Dunnett’s T3 test. Statistical significance is indicated as follows: ^a^ *p* < 0.01, ^b^ *p* < 0.05 vs. intact vehicle, ^c^ *p* < 0.01 vs. PM_2.5_ control (THSD test); ^d^ *p* < 0.01 vs. intact vehicle, ^e^ *p* < 0.01 vs. PM_2.5_ control (DT3 test).

**Table 4:** Lung mRNA expression in intact or PM_2.5_-treated mice.

Groups	Controls		Reference	Test Article—TV		
	Intact Vehicle	PM_2.5_	DEXA	200 mg/kg	100 mg/kg	50 mg/kg
MUC5AC	1.00 ± 0.05	4.96 ± 0.75 ^a^	2.19 ± 0.35 ^ab^	2.46 ± 0.42 ^ab^	2.87 ± 0.27 ^ab^	3.35 ± 0.63 ^ab^
MUC5 B	1.00 ± 0.07	2.85 ± 0.23 ^a^	1.76 ± 0.33 ^ab^	1.85 ± 0.25 ^ab^	2.01 ± 0.22 ^ab^	2.16 ± 0.24 ^ab^
NF-κB	1.00 ± 0.04	8.63 ± 0.89 ^a^	2.09 ± 0.41 ^ab^	3.93 ± 1.10 ^ab^	4.82 ± 1.08 ^ab^	5.87 ± 1.21 ^ab^
p38 MAPK	1.00 ± 0.06	7.27 ± 1.02 ^a^	2.72 ± 0.79 ^ab^	3.79 ± 0.87 ^ab^	4.33 ± 0.80 ^ab^	5.06 ± 0.90 ^ab^
PTEN	1.00 ± 0.06	0.27 ± 0.10 ^a^	0.70 ± 0.10 ^ab^	0.66 ± 0.12 ^ab^	0.60 ± 0.09 ^ab^	0.51 ± 0.05 ^ab^
PI3 K	1.00 ± 0.05	6.34 ± 1.06 ^a^	2.26 ± 0.74 ^ab^	2.69 ± 0.40 ^ab^	3.25 ± 0.75 ^ab^	4.07 ± 0.72 ^ab^
Akt	1.00 ± 0.06	5.09 ± 1.31 ^a^	1.74 ± 0.38 ^ab^	2.06 ± 0.47 ^ab^	2.36 ± 0.45 ^ab^	3.05 ± 0.42 ^ab^
Bcl-2	1.00 ± 0.06	0.38 ± 0.07 ^a^	0.69 ± 0.14 ^ab^	0.60 ± 0.08 ^ab^	0.55 ± 0.08 ^ab^	0.50 ± 0.04 ^ab^
Bax	1.00 ± 0.05	6.93 ± 1.28 ^a^	2.67 ± 0.71 ^ab^	3.56 ± 0.75 ^ab^	3.72 ± 0.83 ^ab^	4.64 ± 0.70 ^ab^

Values represent means ± SD for 10 mice per group. PM_2.5_ refers to Diesel Particulate Matter NIST 1650b; DEXA indicates dexamethasone treatment; TV refers to thyme (*Thymus vulgaris* L.) leaf extract; RT-PCR refers to reverse transcription polymerase chain reaction; NF-κB indicates nuclear factor kappa-light-chain-enhancer of activated B cells; MAPK means mitogen-activated protein kinases; PTEN refers to phosphatase and tensin homolog; PI3K stands for phosphoinositide 3-kinase; Akt denotes protein kinase B; Bcl-2 stands for B-cell lymphoma 2; Bax refers to Bcl-2-associated X protein; THSD denotes Tukey’s Honest Significant Difference test; DT3 indicates Dunnett’s T3 test. Statistical significance is indicated as follows: ^a^ *p* < 0.01 vs. intact vehicle; ^b^ *p* < 0.01 vs. PM_2.5_ control (DT3 test).

**Table 5:** Histomorphometrical analysis of lung—Left lobe tissue in intact or PM_2.5_-treated mice.

Groups	Mean ASA (%/mm^2^)	Mean Alveolar Septal Thickness (μm)	Mean Thickness of SB (μm)	Mean IF Cell Numbers Infiltrated in AR (×10 Cells/mm^2^)	PAS-Positive Cells on the SB	
					Numbers (Cells/mm^2^)	Percentages (%/Epithelium)
Controls						
Intact vehicle	82.66 ± 8.04	4.09 ± 1.46	13.71 ± 1.20	56.80 ± 12.19	24.00 ± 2.98	1.23 ± 0.44
PM_2.5_	38.53 ± 6.10 ^a^	38.32 ± 3.41 ^d^	19.63 ± 1.46 ^d^	883.90 ± 168.40 ^d^	39.60 ± 3.75 ^d^	4.87 ± 0.68 ^d^
Reference						
DEXA	72.16 ± 8.12 ^bc^	12.44 ± 4.89 ^de^	18.95 ± 2.77 ^d^	128.40 ± 31.00 ^de^	38.80 ± 7.44 ^d^	4.65 ± 0.99 ^d^
Test article—TV						
200 mg/kg	67.79 ± 8.16 ^ac^	15.97 ± 2.54 ^de^	28.08 ± 1.83 ^de^	193.60 ± 47.41 ^de^	84.60 ± 10.46 ^de^	20.00 ± 4.00 ^de^
100 mg/kg	60.39 ± 7.96 ^ac^	19.14 ± 3.27 ^de^	25.88 ± 1.45 ^de^	331.00 ± 83.95 ^de^	63.80 ± 10.04 ^de^	12.84 ± 3.25 ^de^
50 mg/kg	54.81 ± 5.57 ^ac^	27.78 ± 4.94 ^de^	23.81 ± 0.77 ^de^	583.00 ± 98.91 ^de^	53.00 ± 6.75 ^de^	7.56 ± 0.90 ^de^

Values represent means ± SD for 10 mice per group. PM_2.5_ refers to Diesel Particulate Matter NIST 1650b; DEXA indicates dexamethasone treatment; TV refers to thyme (*Thymus vulgaris* L.) leaf extract; ASA refers to alveolar surface area; AR indicates alveolar region; SB means secondary bronchus mucosa; IF stands for inflammatory; PAS denotes periodic acid Schiff; THSD refers to Tukey’s Honest Significant Difference test; DT3 indicates Dunnett’s T3 test. Statistical significance is indicated as follows: ^a^ *p* < 0.01, ^b^ *p* < 0.05 vs. intact vehicle, ^c^ *p* < 0.01 vs. PM_2.5_ control (THSD test); ^d^ *p* < 0.01 vs. intact vehicle, ^e^ *p* < 0.01 vs. PM_2.5_ control (DT3 test).

## Data Availability

The original contributions presented in this study are included in the article/Supplementary Materials. Further inquiries can be directed to the corresponding authors.

## Supplementary Materials

The following supporting information can be downloaded at: https://www.mdpi.com/article/10.3390/antiox14111343/s1, Figure S1. Schematic diagram of the manufacturing process for Thymus vulgaris leaf extract; Table S1. Oligonucleotides used for quantitative RT-PCR; Table S2. Body weight gains in intact or PM_2.5_-treated pulmonary-injured mice; Table S3. Lung weights and gross inspections in intact or PM_2.5_-treated pulmonary injured mice.

## Abbreviations

The following abbreviations are used in this manuscript:

ACh	Acetylcholine
ALT	Alanine transaminase
ASA	Alveolar surface area
AST	Aspartate transaminase
BALF	Bronchoalveolar lavage fluid
Bax	BCL2 associated x
Bcl-2	B-cell leukemia/lymphoma 2
CAT	Catalase
CXCL-1	Chemokine (C-X-C Motif) Ligand 1
CXCL-2	Chemokine (C-X-C Motif) Ligand 2
DCFDA	Dichlorodihydrofluorescein diacetate
DEXA	Dexamethasone
ELISA	Enzyme-linked immunosorbent assay
GPx	Glutathione peroxidase
H&E	Hematoxylin and eosin
HO-1	Heme oxygenase-1
IL-6	Interleukin-6
MAPK	Mitogen-activated protein kinase
MDA	Malondialdehyde
MMPs	Matrix metalloproteinases
MUC5AC	Mucin 5AC
MUC5B	Mucin 5B
NF-κB	Kappa-light-chain-enhancer of activated B cells
p38 MAPK	p38 mitogen-activated protein kinase
PAS	Periodic acid-Schiff
PI3K/Akt	Phosphoinositide 3-kinase/Protein kinase B
PM	Particulate matter
PTEN	Phosphatase and tensin homolog
ROS	Reactive oxygen species
SOD	Superoxide dismutase
TNF-α	Tumor necrosis factor-α
TV	Thyme (*Thyme vulgaris* L.) leaf extract
