# Supplementary material for: Protective Effects of Thyme Leaf Extract Against Particulate Matter-Induced Pulmonary Injury in Mice
Source: Antioxidants (Basel). 2025 Nov 7;14(11):1343. doi: 10.3390/antiox14111343 (PMC12649178; doi:10.3390/antiox14111343)
Supplement: Supplementary file 1 [file antioxidants-14-01343-s001.zip › antioxidants-3936520-supplementary.pdf]

Article

Protective Effects of Thyme Leaf Extract Against Particulate Matter-induced Pulmonary Injury in Mice

Jae-Kyoung Lee [1,2,†], Khawaja Muhammad Imran Bashir [3,†], Hye-Rim Park [4], Jin-Gwan Kwon [4], Beom-Rak Choi [4], Jae-Suk Choi [3,*] and Sae-Kwang Ku [5,*]

1 Hongsamdan Co., Ltd., Gongju 32511, Republic of Korea; jklee@hongsamdan.com (J.-K.L.)
2 Department of Food Regulatory Science, College of Science and Technology, Korea University, Sejong 30019, Republic of Korea
3 Department of Seafood Science and Technology, The Institute of Marine Industry, Gyeongsang National University, Tongyeong 53064, Republic of Korea; imranbashir@gnu.ac.kr (K.M.I.B.)
4 Nutracore Co., Ltd., Suwon 16514, Republic of Korea; hrpark@nutracore.co.kr (H.R.P.); jgkwon@nutracore.co.kr (J.G.K.); brchoi@nutracore.co.kr (B.R.C.)
5 Department of Anatomy and Histology, College of Korean Medicine, Daegu Haany University, Gyeongsan 38610, Republic of Korea
* Correspondence: jsc1008@gnu.ac.kr (J.-S.C.); gucci200@dhu.ac.kr (S.-K.K.); Tel.: +82-55-772-9142 (J.-S.C.); +82-53-819-1549 (S.-K.K.)
† These authors contributed equally to this work.

Academic Editor: Firstname Last-name

Received: date
Revised: date
Accepted: date
Published: date

Citation: To be added by editorial staff during production.

Copyright: © 2025 by the authors. Submitted for possible open access publication under the terms and conditions of the Creative Commons Attribution (CC BY) license (https://creativecommons.org/licenses/by/4.0/).

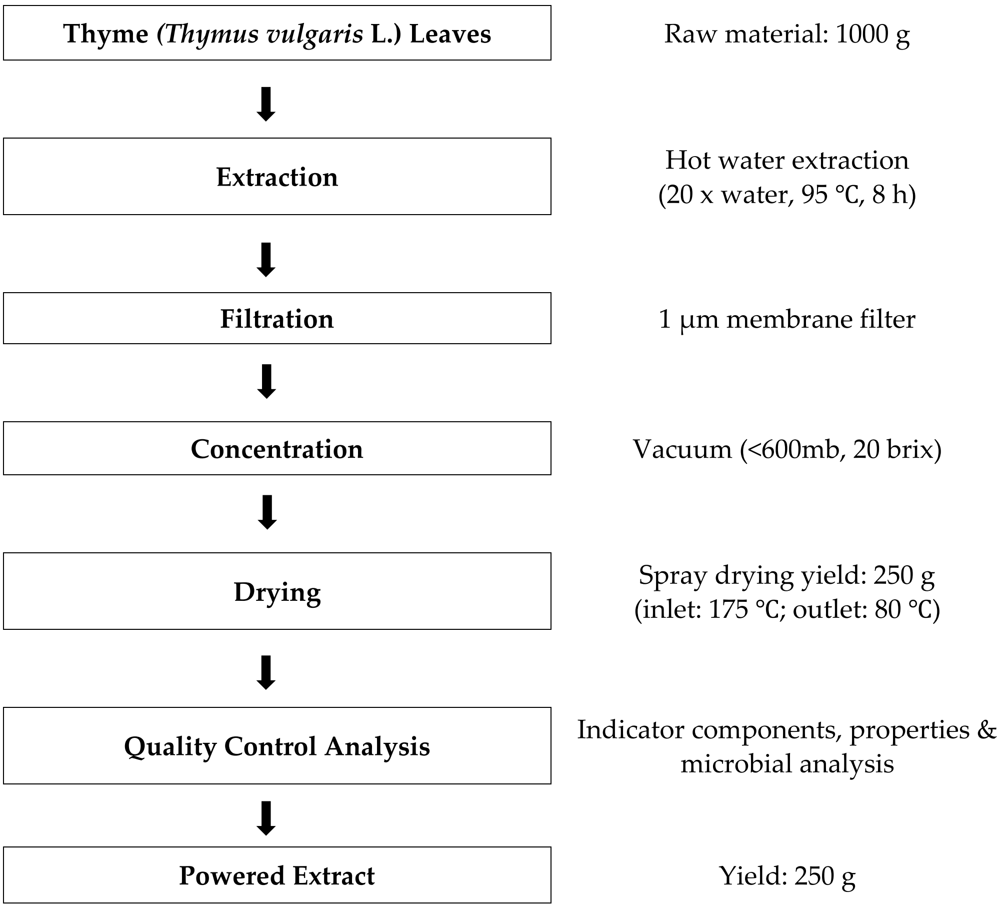

Figure S1. Schematic overview of the *Thymus vulgaris* leaf extract (TV extract) preparation process.

Table S1. Primary sequences used for quantitative RT-PCR.

Target	5′ – 3′	Sequence	NCBI Accession Number
NF-κB	Forward<br>Reverse	CAATGGCTACACAGGACCA<br>CACTGTCACCTGGAACCAGA	NM_008689
PTEN	Forward<br>Reverse	TGGATTCGACTTAGACTTGACCT<br>GCGGTGTCATAATGTCTCTCAG	NM_008960
p38 MAPK	Forward<br>Reverse	CGTTGTTTCCTGGTACAGACC<br>CCATTTCTTCTTGGTCAAGGG	NM_011951
PI3K	Forward<br>Reverse	TCCAAATACCAGCAGGATCA<br>ATGCTTCGATAGCCGTTCTT	NM_008840
Akt	Forward<br>Reverse	TACTCATTCCAGACCCACGA<br>GAGGTTCTCCAGCTTCAGGT	NM_009652
MUC5AC	Forward<br>Reverse	CACCATCTCTACAACCCAAACT<br>TGAGGTCCAGGTCTTTGTGTCT	NM_010844
MUC5B	Forward<br>Reverse	GCCCTCACTGCCTCTGCTCCAC<br>TTTTACAGTGCCAGG GTTTATT	NM_028801
Bcl-2	Forward<br>Reverse	ACCCTGTTGTGTAGCCGTCTG<br>GCATCCCAGCCTCCGTTATCA	NM_009741
Bax	Forward<br>Reverse	GCTTACCGTAGCAGTTGGAT<br>GCCTTGAGCACCAGTTTGCT	NM_007527
β-actin	Forward<br>Reverse	CTGTCGAGTCGCGTCCACCCGCGAG<br>CTCGCGGGTGGACGCGACTCGACAG	NM_007393

RT-PCR represents reverse transcription polymerase chain reaction; NF-κB stands for nuclear factor kappa-light-chain-enhancer of activated B cells; MAPK denotes mitogen-activated protein kinase; PTEN refers to phosphatase and tensin homolog; PI3K indicates phosphoinositide 3-kin ase; Akt signifies protein kinase B; Bcl-2 and Bax correspond to B-cell lymphoma 2 and Bcl-2-associated X protein, respectively.

Table S2. Body weight gains in intact or $PM_{2.5}$-treated mice.

Groups	Body weights at			Body weight gains [B-A]
	Initial test article administration [A]*	Last 10th test article administration	24 h after last 10th test article administration [B]*	
Controls				
Intact vehicle	18.09 ± 0.62	21.83 ± 0.67	18.90 ± 0.58	0.81 ± 0.58
$PM_{2.5}$	18.09 ± 0.73	22.06 ± 0.86	18.91 ± 0.71	0.82 ± 0.79
Reference				
DEXA	17.95 ± 0.85	19.54 ± 0.91[ab]	17.11 ± 0.62[ab]	-0.84 ± 0.34[ab]
Test article – TV				
200 mg/kg	17.95 ± 0.80	21.79 ± 0.56	18.83 ± 0.55	0.88 ± 0.66
100 mg/kg	18.15 ± 0.80	21.82 ± 0.76	18.98 ± 0.77	0.83 ± 0.65
50 mg/kg	18.36 ± 0.85	21.97 ± 0.72	19.18 ± 0.70	0.80 ± 0.54

* All animals were fasted overnight (about 18 h; water was not restricted). Values are expressed as means ± SD of 10 mice. Unit: g; $PM_{2.5}$: Diesel particulate matter NIST 1650b; DEXA: Dexamethasone; TV: Thyme (*Thymus vulgaris* L.) leaf extract; THSD: Tukey's Honest Significant Difference. [a] $p < 0.01$ as compared with intact vehicle control by THSD test; [b] $p < 0.01$ as compared with $PM_{2.5}$ control by THSD test.

Table S3. Lung weights and gross morphological assessment of in intact or $PM_{2.5}$-treated mice.

Groups	Lung weights		Congestional regions (%) – gross findings
	Absolute (g)	Relative (%)	
Controls			
Intact vehicle	0.122 ± 0.007	0.643 ± 0.029	1.51 ± 1.10
$PM_{2.5}$	0.187 ± 0.007^{c}	0.988 ± 0.063^{a}	58.60 ± 13.07^{c}
Reference			
DEXA	0.132 ± 0.007^{de}	0.773 ± 0.048^{ab}	7.20 ± 1.89^{ce}
Test article – TV			
200 mg/kg	0.143 ± 0.013^{ce}	0.760 ± 0.069^{ab}	14.30 ± 5.94^{ce}
100 mg/kg	0.150 ± 0.014^{ce}	0.789 ± 0.074^{ab}	22.00 ± 6.99^{ce}
50 mg/kg	0.160 ± 0.009^{ce}	0.837 ± 0.060^{ab}	35.11 ± 6.72^{ce}

Values are presented as means ± SD (n = 10). $PM_{2.5}$ refers to diesel particulate matter (NIST 1650b); DEXA denotes dexamethasone; TV represents thyme (*Thymus vulgaris* L.) leaf extract; THSD indicates Tukey's Honest Significant Difference; and DT3 corresponds to Dunnett's T3 test. Statistical significance is indicated as follows: ^{a} $p < 0.01$ vs. intact vehicle control, ^{b} $p < 0.01$ vs. $PM_{2.5}$ control (THSD test); ^{c} $p < 0.01$ and ^{d} $p < 0.05$ vs. intact vehicle control, ^{e} $p < 0.01$ vs. $PM_{2.5}$ control (DT3 test).
